# TDP-43 regulates LC3ylation in neural tissue through ATG4B cryptic splicing inhibition

**DOI:** 10.1007/s00401-024-02780-4

**Published:** 2024-09-21

**Authors:** Pascual Torres, Santiago Rico-Rios, Miriam Ceron-Codorniu, Marta Santacreu-Vilaseca, David Seoane-Miraz, Yahya Jad, Victòria Ayala, Guillermo Mariño, Maria Beltran, Maria P. Miralles, Pol Andrés-Benito, Joaquin Fernandez-Irigoyen, Enrique Santamaria, Carlos López-Otín, Rosa M. Soler, Monica Povedano, Isidro Ferrer, Reinald Pamplona, Matthew J. A. Wood, Miguel A. Varela, Manuel Portero-Otin

**Affiliations:** 1 Metabolic Pathophysiology Research Group, Department of Experimental Medicine, University of Lleida (UdL), Lleida Biomedical Research Institute (IRBLleida), 25198 Lleida, Spain; 2 Department of Paediatrics, Institute of Developmental and Regenerative Medicine (IDRM), University of Oxford, Roosevelt Dr, Oxford, OX3 7TY UK; 3 MDUK Oxford Neuromuscular Centre, University of Oxford, Oxford, UK; 4 Departamento de Biología Funcional, Facultad de Medicina, Universidad de Oviedo, 33006 Oviedo, Spain; 5 Instituto Universitario de Oncología (IUOPA), 33006 Oviedo, Spain; 6 Instituto de Investigación Sanitaria Del Principado de Asturias (ISPA), 33011 Oviedo, Spain; 7 Neuronal Signaling Unit, Department of Experimental Medicine, Lleida Biomedical Research Institute (IRBLleida), University of Lleida (UdL), 25198 Lleida, Spain; 8 Neurologic Diseases and Neurogenetics Group, Bellvitge Institute for Biomedical Research (IDIBELL), 08907 L’Hospitalet de Llobregat, Spain; 9 CIBERNED (Network Centre of Biomedical Research of Neurodegenerative Diseases), Institute of Health Carlos III, 08907 L’Hospitalet de Llobregat, Barcelona Spain; 10 Clinical Neuroproteomics Unit, Proteomics Platform, Proteored-ISCIII, Navarrabiomed, Complejo Hospitalario de Navarra (CHN), Universidad Pública de Navarra (UPNA), diSNA, 31008 Pamplona, Spain; 11 Departamento de Bioquímica y Biología Molecular, Universidad de Oviedo, 33006 Oviedo, Spain; 12 Neuropathology Group, Institute of Biomedical Research, IDIBELL, 08907 L’Hospitalet de Llobregat, Spain; 13 Department of Pathology and Experimental Therapeutics, University of Barcelona, 08007 Barcelona, Spain

**Keywords:** ALS, Antisense oligonucleotides, Autophagy, Digital PCR, Post-translational modification

## Abstract

Amyotrophic lateral sclerosis (ALS) is an adult-onset motor neuron disease with a mean survival time of three years. The 97% of the cases have TDP-43 nuclear depletion and cytoplasmic aggregation in motor neurons. TDP-43 prevents non-conserved cryptic exon splicing in certain genes, maintaining transcript stability, including *ATG4B*, which is crucial for autophagosome maturation and Microtubule-associated proteins 1A/1B light chain 3B (LC3B) homeostasis. In ALS mice (G93A), *Atg4b* depletion worsens survival rates and autophagy function. For the first time, we observed an elevation of LC3ylation in the CNS of both ALS patients and *atg4b*^*−/−*^ mouse spinal cords. Furthermore, LC3ylation modulates the distribution of ATG3 across membrane compartments. Antisense oligonucleotides (ASOs) targeting cryptic exon restore *ATG4B* mRNA in *TARDBP* knockdown cells. We further developed multi-target ASOs targeting TDP-43 binding sequences for a broader effect. Importantly, our ASO based in peptide-PMO conjugates show brain distribution post-IV administration, offering a non-invasive ASO-based treatment avenue for neurodegenerative diseases.

**Supplementary Information:**

The online version contains supplementary material available at 10.1007/s00401-024-02780-4.

## Introduction

Amyotrophic lateral sclerosis (ALS) is the most common human motor neuron disease in adults and is characterised by a progressive paralysis and muscular atrophy. Life expectancy after diagnosis ranges from two to five years. Riluzole is the only drug approved for ALS in the European Union (EU), which prolongs survival by two to six months. The cause of ALS is unknown, although 5–10% of the patients have a dominant inheritance of the disease. This form is known as familial ALS (fALS). Different genes associated with RNA metabolism, oxidative stress, and autophagy, among others, are found mutated in fALS. On the other hand, in 90–95% of ALS cases there is not known family history of the disease. This form corresponds to sporadic ALS (sALS) [11]. The spinal cord (SC) of sALS patients is characterised by the presence of TDP-43 aggregates and nuclear clearance [32]. TDP-43 is the protein product of the TAR DNA-Binding Protein 43 (*TARDBP)* gene and is also mutated in fALS [11]. TDP-43 is a mainly nuclear RNA binding protein involved in RNA metabolism, including pre-mRNA splicing. In 2015, Ling et al., discovered a new set of non-conserved cryptic exons that are repressed by TDP-43, including one in ATG4B in human but not in mice, limiting the studies in pre-clinical models [28]. When TDP-43 function is compromised, cryptic exons are spliced in mRNA, which can introduce frame-shift mutations, triggering a downregulation of the mRNA by non-sense mRNA-mediated decay (NMD) [17].

We have already shown that clearance of TDP-43 from the nucleus leads to cryptic exon splicing and mRNA degradation of ATG4B (an autophagy protein), decreasing the clearance of protein aggregates associated with neurodegeneration [47]. Notably, ATG4B regulates SQSTM1 accumulation [53]. SQSTM1 aggregates are a common hallmark in diseased motor neurons in ALS and an impaired clearance is associated with a shorter survival [37].

In the same line, our group have demonstrated that high *ATG4B* cryptic exons levels are correlated with disease duration [47] and advanced Braak stages in AD [44], suggesting a pathological role in TDP-43 proteinophaties.

However, *Atg4b* genetic deletion in mice (*atg4b*^*−/−*^) only have mild symptoms affecting the sense of balance [30]. It can be explained because an autophagy stress (commonly found in ALS affected tissue) [6] is necessary to make this protein essential for cell survival [33]. Moreover, ATG4B function on SQSTM1 clearance is not compensated by any of the other ATG4 homologues [33]. Furthermore, enzymatic parameters of ATG4B greatly outperform those from the other ATG4 cysteine-proteases homologues processing ATG8 substrates, highlighting its special relevance in autophagy flux [26]. ATG8 substrates include Microtubule-associated proteins 1A/1B light chain 3B (LC3B) in mammals. LC3B was firstly described as a microtubule associated protein [29]. Later, a crucial role of LC3B in autophagy was discovered. Firstly, ATG4 proteins cleavage pro-LC3B, producing LC3B-I. Then, LC3B-I is lipidated, resulting in LC3B-II which is incorporated into autophagosome membranes. SQSTM1 recognises ubiquitin-tagged proteins and drivers their location into the autophagosome by tethering LC3B, thus triggering their autophagic degradation [24].

ATG4B has another function independent of autophagy that has been recently demonstrated only in vitro [1]. This protein can remove LC3B conjugates (LC3ylation) covalently bound to other proteins in a process called deLC3ylation. Little is known about this post-translational modification, which was only observed after overexpression of truncated LC3B in *ATG4B* KO HeLa cells [1]. ATG3 is one of the LC3ylated proteins. However, the consequences of this modification are unknown. LC3B can also modify other proteins by a reversible thioester, such as ATG3 and ATG7 partners during the formation of the autophagosome [12]. Unlike thioester binding, LC3ylation is not removed with the addition of a reducer like β-mercaptoethanol. Little is known about LC3ylation targets and function, potentially linked to mitophagy [5]. We hypothesise that ATG4B dysregulation could compromise cell survival in a stress situation due to a lack of autophagy and deLC3ylation activity, becoming an excellent potential target for ALS therapy.

A useful approach to target a single splicing event is the use of antisense oligonucleotides (ASOs). The development of such precision medicines to treat human neuromuscular disease represents a major paradigm shift in medical science. Of note is the approval of the intrathecal administration of Nusinersen [8] for the treatment of spinal muscular atrophy (SMA), a disease linked to a homozygous deletion of *SMN1* exon 7. Approximately 10% of the mRNA from *SMN2* contains exon 7 and, thus, can generate some functional, full-length SMN protein. This allows for partial compensation of the lost *SMN1* exon 7 by *SMN2* synthesis [7].ASO chemistry are a relevant factor for biodistribution, efficacy and toxicity. Therefore, the research on this field has gained interest in the recent years. For example, Locked Nucleic Acid (LNA) mixmers are more efficient than classical MOE chemistry of Nusinersen inducing *SMN1* exon 7 splicing [48]. Notably, Peptide conjugation of cell-penetreating peptides (CPP) in Peptide Phosphorodiamidate Morpholino Oligomer (P-PMO) ASOs enhances the delivery into cells [20, 55]. Moreover P-PMO chemistry is also useful to modulate biodistribution. Although Nusinersen has been the first success to treat SMA, intrathecal (IT) administration results in an invasive procedure with potential severe side effects. P-PMO could overcome that problem by enriching the concentration in nervous tissue by intravenous (IV) administration.

In the case of ALS, different ASOs strategies are being studied. In April 2023, FDA approved Tofersen an ASO that mediates RNase H-dependent degradation of superoxide dismutase 1 (SOD1). A phase 3 clinical trial is still ongoing for Tofersen to confirm clinical benefit. ION363 is an antisense oligonucleotide designed to reduce the production of a mutated, neurotoxic form of the Fused in Sarcoma (FUS) protein. ION363 (NCT04768972) showed clinical benefits in a FUS mouse model and a tendency toward ALSFRS-R score stabilization upon ION363 treatment in one patient [25]. Nevertheless, both Tofersen and ION363 are only useful for a few subtypes of fALS, accounting for a very limited number of ALS patients. In contrast, cryptic splicing is found in 97% of patients (including sALS) and is typically a disease-specific event, preserving the correct splicing pattern in non-diseased cells, in contrast to Tofersen and ION363 that also downregulate normal genes. However, hundreds of genes are predicted to have a cryptic exon [28]. Determining the set of major disease-modifying cryptic exons is critical to design targeted therapy.

The present study is designed for the development of ASOs to inhibit *ATG4B* cryptic splicing. We show that ATG4B autophagic and deLC3ylating function is compromised in human and mouse ALS samples. Moreover, our LNA mixmer ASOs and a novel P-PMO with brain distribution within IV administration restore *ATG4B* expression in ALS-linked models, becoming a promising therapy for sALS.

## Materials and methods

### Human samples

All samples were obtained from the Institute of Neuropathology and the University of Barcelona Brain Bank following the guidelines of the local ethics committees. Extensive pathological studies were conducted for ALS diagnosis as previously described [19] (Table 1). Briefly, all ALS patients were free from a familial history of ALS, and frontotemporal dementia symptoms and signs. Further, they were evaluated for known ALS-related mutations in *C9orf72*, *SOD1*, *TARDBP*, *ATX2*, *FUS* and *UNC13A* genes, which were not found in the present series. Therefore, all samples evaluated were considered as sporadic ALS patients.

**Table 1:** Demographics of samples used in this study

ID	Sample type	Diagnosis^a^	Sex	Age	PM delay
1	Frontal Cortex	Control	M	61	7 h 45 m
2	Frontal Cortex	Sporadic ALS	F	57	8 h
3	WBC	Control	M	60	–
4	WBC	Control	M	68	–
5	WBC	Control	F	66	–
6	WBC	Control	M	N/A	–
7	WBC	Control	M	74	–
8	WBC	Control	F	N/A	–
9	WBC	Control	F	76	–
10	WBC	Control	M	67	–
11	WBC	Control	F	72	–
12	WBC	Control	F	44	–
13	WBC	Control	F	66	–
14	WBC	Control	F	63	–
15	WBC	Sporadic ALS	M	60	–
16	WBC	Sporadic ALS	M	63	–
17	WBC	Sporadic ALS	F	66	–
18	WBC	Sporadic ALS	F	53	–
19	WBC	Sporadic ALS	M	73	–
20	WBC	Sporadic ALS	M	65	–
21	WBC	Sporadic ALS	F	43	–
22	WBC	Sporadic ALS	F	57	–
23	WBC	Sporadic ALS	F	N/A	–
24	WBC	Sporadic ALS	M	73	–
25	WBC	Sporadic ALS	F	N/A	–
26	WBC	Sporadic ALS	M N/A	N/A N/A	–
27	Spinal cord	Control	M	56	07 h 10 min
28	Spinal cord	Control	F	64	11 h 20 min
29	Spinal cord	Control	M	80	4 h 20 min
30	Spinal cord	Control	M	66	14 h
31	Spinal Cord	Control	F	75	6 h 10 min
32	Spinal cord	Sporadic ALS	F	79	02 h 10 min
33	Spinal cord	Sporadic ALS	F	57	4 h
34	Spinal cord	Sporadic ALS	F	57	10 h
35	Spinal cord	Sporadic ALS	F	75	4 h 05 min
36	Spinal cord	Sporadic ALS	M	69	02 h
37	Spinal cord	Sporadic ALS	M	64	16 h 30 min
38	Spinal cord	Sporadic ALS	M	54	4 h 50 min
39	Spinal cord	Sporadic ALS	F	76	13 h
40	Spinal cord	Sporadic ALS	F	83	15 h 15 min
41	Spinal cord membrane-rich fraction	Control	M	66	4 h 55 min
42	Spinal cord membrane-rich fraction	Control	F	60	09 h 40 min
43	Spinal cord membrane-rich fraction	Control	M	52	3 h
44	Spinal cord membrane-rich fraction	Control	M	61	3 h 55 min
45	Spinal cord membrane-rich fraction	Sporadic ALS	M	46	7 h
46	Spinal cord membrane-rich fraction	Sporadic ALS	F	69	17 h
47	Spinal cord membrane-rich fraction	Sporadic ALS	F	68	16 h 30 min
48	Spinal cord membrane-rich fraction	Sporadic ALS	F	63	19 h
49	Spinal cord membrane-rich fraction	Sporadic ALS	F	63	13 h 50 min
50	Frontal cortex	Control	M	66	18 h 00 min
51	Frontal CORTEX	Control	M	61	03 h 40 min
52	Frontal cortex	Control	M	62	05 h 45 min
53	Frontal cortex	Control	M	74	06 h 40 min
54	Frontal cortex	Control	M	65	05 h 15 min
55	Frontal cortex	Control	F	64	02 h 15 min
56	Frontal cortex	Control	M	63	08 h 05 min
57	Frontal cortex	Control	F	79	03 h 35 min
58	Frontal cortex	Control	F	67	05 h 20 min
59	Frontal cortex	Control	M	70	03 h 45 min
60	Frontal cortex	Control	M	52	04 h 40 min
61	Frontal cortex	Control	F	52	05 h 45 min
62	Frontal cortex	Control	F	82	07 h 35 min
63	Frontal cortex	Control	F	74	02 h 45 min
64	Frontal cortex	Control	M	55	5 h 40 min
65	Frontal cortex	Control	M	59	7 h 05 min
66	Frontal cortex	Control	M	56	3 h 50 min
67	Frontal cortex	Sporadic ALS	M	70	3 h 00 min
68	Frontal cortex	Sporadic ALS	F	56	3 h 45 min
69	Frontal cortex	Sporadic ALS	M	59	3 h 15 min
70	Frontal cortex	Sporadic ALS	F	63	13 h 50 min
71	Frontal cortex	Sporadic ALS	F	59	14 h 15 min
72	Frontal cortex	Sporadic ALS	M	54	4 h 50 min
73	Frontal cortex	Sporadic ALS	M	76	12 h 40 min
74	Frontal cortex	Sporadic ALS	M	64	16 h 30 min
75	Frontal cortex	Sporadic ALS	F	57	4 h 00 min
76	Frontal cortex	Sporadic ALS	F	75	4 h 05 min
77	Frontal cortex	Sporadic ALS	F	57	10 h 00 min
78	Frontal cortex	Sporadic ALS	M	50	10 h 10 min
79	Frontal cortex	Sporadic ALS	F	59	2 h 30 min
80	Frontal cortex	Sporadic ALS	M	46	7 h 00 min
81	Frontal cortex	Sporadic ALS	F	69	17 h 00 min

^a^ All samples included were tested for known *C9orf72*, *SOD1*, *TARDBP*, *ATX2*, *FUS* and *UNC13A* mutations, which were not found in any of cases (being thus considered sporadic ALS). *M* male. *F* female. *ALS* amyotrophic lateral sclerosis. *PM* Post-Mortem. *WBC* whole blood cells

### RT-qPCR

RNA was extracted from cells using TRI Reagent (Thermo Fisher Scientific, AM9738) following the manufacturer’s instructions. RNA concentrations were measured using a NanoDrop ND-1000 (Thermo Fisher Scientific). One microgram of RNA was used for retrotranscription utilizing TaqMan Reverse Transcription Reagent using random hexamers (Thermo Fisher Scientific, N8080234).

Cryptic exons were quantified as previously described [44, 47] (Table 2). Briefly, RT-qPCR experiments were performed using a CFX96 instrument (Bio-Rad) with SYBR Select Master mix for CFX (Thermo Fisher Scientific, 4472937). Each 20 µL reaction contained 4µL cDNA, 10 µL SYBR Select Master Mix, 0.2 nM of the forward primer and 0.2 nM of the reverse primer solutions, as well as 4 µL PCR grade water. RT-qPCR run protocol was as follows: 50 °C for 2 min and 95 °C for 2 min, with the 95 °C for 15 s and 60 °C for 1-min steps repeated for 40 cycles; and a melting curve test from 65 to 95 °C at a 0.1 °C/s measuring rate. Primers employed in these experiments are listed in Table 2. Cryptic exon inclusion or Percentage Spliced-In (PSI) was estimated using the following formula: 100 × 2^(−Conserved exon Cq –Cryptic exon Cq)^.

**Table 2:** Sequences of primers used in PCRs performed in this study

Genes	Forward	Reverse
TOTAL ATG4B	5′-AACGCATTCATCGACAGGAAG-3′	5′-TTTGCGCTATCTGGTGAATGG-3′
CRYPTIC ATG4B	5′-CTGAGTGTGCATGGATGAGTG-3′	5′-TTGCTGGCACCAATCATTGAA-3′
TOTAL GPSM2	5’-GGACGTGCCTTTGGAAATCTT-3′	5′-TTTGCAATAAGGAGACGCTGC-3’
CRYPTIC GPSM2	5′-GTGTGTATGAGAGAGAGAGCGA-3′	5′-AGAAGCTTCCATTCTGTTCATCA-3′
TOTAL PFKP	5′-GACCTTCGTTCTGGAGGTGAT-3′	5′-CACGGTTCTCCGAGAGTTTG-3′
CRYPTIC PFKP	5′-ACGTTTGCAAAACATCAGGAG-3′	5′-GCCTTCAACTCTCCGTTCAC-3′
TARDBP	5′-CTGCGGGAGTTCTTCTCTCA-3’	5′-CGCAATCTGATCATCTGCAA-3’
GAPDH	5′-CCCTTCATTGACCTCAACTACATG-3′	5′-TGGGATTTCCATTGATGACAAG-3′

In the case of frontal cortex specimens, frozen samples of the area 8 (n = 15 sALS and n = 17 controls) were obtained for RNA extraction using RNeasy Mini Kit (Qiagen, 74104) following the instructions of the supplier. RNA concentration was evaluated using a NanoDrop™ Spectrophotometer (Thermo Fisher Scientific). Complementary DNA (cDNA) was prepared using High-Capacity cDNA Reverse Transcription kit (Thermo Fisher Scientific, 4368814) following the protocol provided by the supplier. Parallel reactions for each RNA sample were run in the absence of MultiScribe Reverse Transcriptase to assess the lack of contamination of genomic DNA. TaqMan RT-qPCR assays were performed in duplicate for each gene on cDNA samples in 384-well optical plates using an ABI Prism 7900 Sequence Detection system (Thermo Fisher Scientific). For each 5 μL TaqMan reaction, 2.25 μL cDNA was mixed with 0.25 μL 20 × TaqMan Gene Expression Assays and 2.50 μL of 2 × TaqMan Universal PCR Master Mix (Thermo Fisher Scientific, 4304437). Analyzed genes included the human *TARDBP* TaqMan probe (Hs00606522_m1). The mean value of one house-keeping gene, β‐glucuronidase (GUS‐β) (Hs00939627_m1) was used as internal control for normalization in frontal cortex samples. The selection of GUS-β as a house-keeping genes was due to our previous experience noting that other markers such as β-actin, tubulin, β-glucuronidase (GUS), superoxide dismutase 1 (SOD1), and metalloproteinase domain 22 (ADAM22) mRNAs had disparate expression in the human post-mortem control nervous tissue. The parameters of the reactions were 50 °C for 2 min, 95 °C for 10 min, and 40 cycles of 95 °C for 15 s and 60 °C for 1 min. Finally, the capture of all TaqMan PCR data was with the Sequence Detection Software (Thermo Fisher Scientific, SDS version 2.2.2). The double-delta cycle threshold (ΔΔCT) method was used to analyze the data.

### Digital PCR (dPCR)

QIAcuity One 2plex Digital PCR System (QIAGEN) was used for dPCR experiments. QIAcuity OneStep Advanced Probe Kit (QIAGEN, 250131) was employed for absolute quantification of ATG4B mRNA molecules in samples following the manufacturer’s instructions. Briefly, 500 ng of RNA from whole blood cells (12 controls 12 ALS cases) or frontal cortex (1 control 1 ALS patient) were mixed with one-step reagents and the following set of primers (Sigma-Aldrich):

ATG4B-cryptic-Fwd: GTCCATCGCTGTGCATGTTG

ATG4B-cryptic-Rev: CCATGAAGGCTGCACAGGA

ATG4B-cryptic-probe: FAM-TCACGTGGTTGGGAATCTGAAGGG-BHQ-1

ATG4B-total-Fwd: TTGCTGTCTTCGATACGTGG

ATG4B-total-Rev: GGTCGGAATCTGCAGGAAAC

ATG4B-total-probe: HEX-TGGAGGAAATCAGAAGGTTGTGCAGG-BHQ-1

Cycling protocol was: an initial step for reverse transcription of 40 min at 50 °C, PCR initial heat activation for 2 min at 95 °C, 2-step cycling (40 cycles) composed of denaturation for 5 s at 95 °C and combined annealing/extension for 30 s at 60 °C. The plate was the imaged and analysed with QIAcuity Software Suite 1.2.18 with an automated threshold for positive/negative partition discrimination. Results were displayed as copies/μl of loaded RNA.

### Oligonucleotide synthesis

The cell-penetrating peptide (CPP) Pip8b2 was used to aid oligonucleotide delivery. This CPP contains two flanking regions enriched with cationic amino acids and a central hydrophobic core: N-terminus (Ac), Left Domain (RXRRBRR), Hydrophobic Core (YQFLI), Right Domain (RBRXR), Linker (B), C-terminus (PMO: TCAGATTCCCAACCACGTGAACACA). Amino acids were L stereoisomers, except for the non-natural B and X which have no side chains. P-PMO are non-ionic oligonucleotides synthesized by replacing the phosphodiester bond by a phosphoramidate linkage and the ribose by a morpholino moiety. PMOs were purchased from Gene Tools LLC. Conjugation of PMO and peptide by covalent bond is followed by centrifugation through 3 k Amicon filters and filtration through 0.22 mm. Quality control is performed after each of the conjugations (high-performance liquid chromatography [HPLC].: > 99%; MALDI-TOF: an acceptance error of molecular weight is 0.1%). LNAs were purchased from IDT, all oligonucleotide sequences are shown in Table 3.

**Table 3:** Sequences of the antisense oligonucleotides used to modify the splicing of TDP-43 target genes

ASO	Sequence
CA	+ C*A*C* + A*C*A* + C*A*C* + A*C*A* + C*A*C* + A
CATA	+ C*A*C* + A*C*A* + T*A*C* + A*C*A* + C*A*C* + A
ACNN	+ C*A*C* + A*C*A* + C*N*N* + A*C*A* + C*A*C* + A
5U	+ C*A*A* + A*C*A* + G*C*A* + T*T*C* + A*G*C* + A
5	+ C*A*A* + C*C*A* + C*G*T* + G*A*A* + C*A*C* + A
3 J	+ A*C*A* + C*A*C* + T*C*A* + C*C*A* + T*G*G* + C
Pip8b2-ATG4B	Ac-RXRRBRR YQFLI RBRXRB-pmo-TCAGATTCCCAACCACGTGAACACA
SCR	+ C* + A* + T*G*T*A*C*T*C*A*A*C*C* + T* + C* + A

+ represents Locked Nucleic Acids and *phosphorothiate linkages

### Animal experiments

A colony of the strain B6.Cg-Tg (SOD1*G93A)1Gur/J (JAX, 004435) was maintained in C57BL/6 J background. *Atg4b* KO mice were previously described[30] After genotyping and weaning, animals were placed at 12:12 h dark/light cycle, at 22 ± 2 °C temperature, 50% ± 10 relative humidity, in individual cages (at 21 days old). This study was approved by the Animal Research and Ethics Committee at the University of Lleida. Animals were weighed weekly. Cervical dislocation was employed to euthanize the animals at the clinical endpoint (righting reflex > 20 s) or 180 days for non-transgenic mice.

Tissues were rapidly excised, snap-frozen in liquid N_2_, and stored at− 80 °C. All experimental procedures were approved by the Institutional Animal Care Committee of the University of Lleida, in compliance with local laws and pursuant to the Directive 2010/63/EU of the European Parliament. Experiments performed in the UK were authorized and approved by the University of Oxford ethics committee and UK Home Office (project license 30/2907).

### Biodistribution

The concentration of oligonucleotides in mouse tissue was measured by custom ELISAs in brain, kidney, liver, heart, gastrocnemius, and quadriceps. The ELISA was performed as described in [4] using the following phosphorothioate probe: (5′- > 3′) [DIG]. T*G*T*G*T*T*C*A*C*GTGGTTG*G*G*A*A*T*C*T*G*A [BIO], which is double-labelled with digoxigenin and biotin.

### Cell culture and treatments

#### HeLa cells

HeLa cells were maintained in Dulbecco's Modified Eagle's Medium (DMEM) (Thermo Fisher Scientific, 11965), 10% FBS (Thermo Fisher Scientific, 10270), 100 U/ml Penicillin–Streptomycin (Thermo Fisher Scientific, 15140–122) at 37 °C and 5% CO_2_. 150,000 HeLa cells were seeded in a 6-well plate and transfected with 20 nM of *TARDBP* siRNA (SIGMA, EHU109221) mixed with 2 µl RNAiMAX (Thermo Fisher Scientific, 13778100) in 100 µl Opti-MEM (Thermo Fisher Scientific, 31985062). After 24 h, cells were transfected with LNA mixmers at 5, 10, 20, 40, or 100 nM mixed with 1 µl of RNAiMAX per 10 nM of ASO in 100 µl Opti-MEM. P-PMO was sonicated for 10 min at 37 °C an added directly to the cell media at 10 or 20 µM.

TDP-43 splicing reporter (pHBS1389 IBB-GFP-mCherry3E) was a gift from Rajat Rohatgi (Addgene plasmid # 118,803; http://n2t.net/addgene:118803; RRID:Addgene_118803) [38]. TDP-43 silenced HeLa cells were transfected with 2 µg mixed with 2 µl of Lipofectamine 3000 and P300 (Thermo Fisher Scientific, L3000001) in 100 µl Opti-MEM (Thermo Fisher Scientific, 31985062). In parallel, cells were transfected with ASO CA 400 nM. Fluorescence was checked 24- and 48-h post-transfection. Fluorescence intensity was measured with CellProfiler [43] for RFP and GFP and the ratio was estimated as TDP-43 function level.

#### Mouse fibroblasts

Skin primary fibroblasts were obtained from the ears of 4-month-old mice. Ears were chopped into small fragments and rinsed with ADS buffer. These fragments were digested with 0.2 U/ml of type 2 collagenase (Worthington Biochemical Corporation, CLS-2) in 1 ml of ADS. Samples were shaken at 37 °C for 45 min. Supernatant was transferred to a sterile tube with complete DMEM 10% FBS to stop the reaction. This step was repeated two times. The tube containing isolated fibroblasts was centrifuged at 300 RCF. Pelleted cells were seeded in a 100 mm culture plate and maintained in DMEM 10% FBS, 100 U/ml Penicillin–Streptomycin at 37 °C and 5% CO_2_.

For autophagy response, 100,000 fibroblasts from WT, *atg4b*^−/−^, G93A, or G93A *atg4b*^−/−^ mice were seeded in a 6 well plate. Cells were treated with chloroquine (Sigma-Aldrich, C6628) 30 µM or incubated with HBSS (Thermo Fisher Scientific, 14025) for 24 h.

#### Human iPSC motor neurons

Human iPSC Motor neurons were generated as previously described [35] Two hours after seeding 100,000 cells from disaggregated neurospheres in 35 mm well plate, 200 µl of cell medium containing lentiviruses were added with 500 µl of motor neuron induction media. After 24 h, cell media was refreshed. Cells were harvested after 6 days.

#### Lentivirus production

Three million HEK293T cells were seeded in 100 mm well plate 24 h prior transfection. Transfection protocol was performed following manufacturer instructions. Briefly, 13 µg of psPAX2 (Addgene, 12260), 7 µg pMD2.G (Addgene, 12259) both a gift from Dr. Trono) and 20 µg of pLVTHM plasmid [52] containing shRNA against *TARDBP* or scrambled [47] were mixed by vortex with 120 µl of Fugene HD (Promega, E2311) in 1 ml OMEM (Thermo Fisher Scientific, 31985062) and incubated at room temperature for 15 min. Then, the transfection mix was added to HEK293T cells. After 24 h of incubation at 37 °C with 5% CO_2_, cell medium was removed and refreshed with 10 ml of fresh medium and incubated for 72 h. Cell medium was collected and stored at − 80 °C.

### Western blot analyses

Protein from cells was extracted with 100 µL of radioimmunoprecipitation (RIPA) buffer with protease inhibitor (Thermo Fisher Scientific, 78429) and phosphatase inhibitor (1 mM NaF and 1 mM Na_3_VO_4_). Protein from tissue was extracted homogenizing the sample in a buffer containing 180 mM KCl, 5 mM MOPS, 2 mM EDTA, 1 mM diethylenetriaminepentaacetic acid at pH 7 with protease and phosphatase inhibitors. After sonication, protein quantification was performed with Bradford assay (Bio-Rad, 5000006). 15 μg of protein in reducing Laemmli buffer (60 mM Tris–HCl pH 6.8; 20% glycerol; 2% SDS; 4% beta-mercaptoethanol; 0.01% bromophenol blue) were loaded onto a 12% acrylamide SDS-PAGE gel. Membranes were blocked with I-Block (Thermo Fisher Scientific, T2015) for 1 h and incubated overnight with primary antibody (Table 4) in TBS-T 0.05%. After primary antibody incubation, membranes were washed 3 times with TBS-T 0.05% and incubated with secondary antibody for 1 h. Immobilon™ Western Chemiluminiscent HRP Substrate (Merck Millipore, WBKLS0500) was used for immunodetection. Membranes were stained with Coomassie Brilliant Blue G (Sigma-Aldrich, 27815) for normalization. In the case of human iPSC motor neurons, protein expression was normalised with actin expression due to low Coomassie staining. Specific bands were quantified with ImageLab v5.2.1 (Bio-Rad).

**Table 4:** List of primary antibodies and hybridization conditions used in this study

Target	Western Blot dilution	IF/IHQ dilution	IP (µg of antibody)	Manufacturer	Catalogue number
TDP-43	1000	–	–	Proteintech	10782–2-AP
ATG4B	500	200	–	Sigma	A2981
LC3B (WB)	1000	–	–	Cell Signaling Technology	2775
LC3B (IP)	–	–	5	Cell Signaling Technology	83506
SQSTM1	1000	–	–	Cell Signaling Technology	5114
Mouse IgG1 kappa isotype control (IP)	–	–	5	Thermo Fisher Scientific	14–4714-81
GFAP	1000	–	–	Abcam	ab7260
HB9	1000	–	–	Abcam	ab92606
ATG3	1000	–	–	Proteintech	11262–2-AP
Actin	1000		–	Abcam	ab20272

### Immunohistochemistry

Three control donors and three ALS fixed paraffin embedded SC slides were dried for 1 h at 65° before the pre-treatment procedure of deparaffinization, rehydration and epitope retrieval in the Pre-Treatment Module (Agilent Technologies-DAKO, PT-LINK) at 95 °C for 20 min in 50 × Tris/EDTA buffer, pH 9. After incubation with anti-ATG4B (Table 4), the reaction was visualized with the EnVisionTM FLEX Detection Kit (Agilent Technologies-DAKO) using diaminobenzidine chromogen as a substrate Sections were counterstained with hematoxylin.

### Immunoprecipitation

LSCs were extracted from WT and *Atg4b* KO mice and homogenized with RIPA buffer. 500 µg were used for the experiments. We employed Dynabeads™ Protein G (Thermo Fisher Scientific, 10003D) and followed the manufacturer’s instructions. Briefly, 5 µg of anti-LC3B (Cell Signaling Technologies, 83506) or 5 µg for IP CTL of mouse anti-IgG1kappa isotype Control (Thermo Fisher Scientific, 14–4714-81) were conjugated to Dynabeads Protein G for 1 h at room temperature. Conjugated beads were incubated with LSC homogenates under rotatory agitation for 1 h at room temperature. Beads were then washed 4 times with RIPA buffer and eluted with electrophoresis buffer LB (containing SDS and beta-mercaptoethanol) after heating at 70 °C for 10 min.

### Membrane isolation

Membranes from SC were isolated as described previously [31], with a few modifications. Briefly, tissue was chopped and homogenized with a teflon-pestle grinder. This sample was centrifuged at 600 xg for 10 min at 4 °C 10 min to remove the cell debris. The supernatant was centrifuged at 10,000 xg for 20 min at 4 °C to obtain the crude mitochondria (CM) fraction. This fraction was incubated with Triton X-100 for 1 h at 4 °C on a wheel. This sample was then loaded onto a discontinuous sucrose gradient and ultracentrifuged at 100,000 xg for 16 h at 4 °C. 200 μl of the resulting sample were collected sequentially from the top to the bottom of the tube, corresponding to Fraction 1 to Fraction 25. These fractions were prepared for western blot analyses.

### Polysome isolation

HeLa cells were silenced for 96 h. Cells underwent incubation with cycloheximide (100 μg/ml, 15 min), followed by the generation of cytoplasmic lysates employing PEB buffer. The lysates were subjected to size-fractionation through 10–50% sucrose gradients in a centrifuge, yielding 12 fractions that were subsequently collected for further examination [36]. The distribution of *ATG4B* (total and cryptic) mRNA across the gradient was scrutinized through RT-qPCR analysis, and the data were depicted as the proportion of each specific mRNA in relation to the quantity of that mRNA in the last fraction of the non-treated cells or the Percentage Spliced-in (PSI) of cryptic exon inclusion in the total *ATG4B* mRNA.

### Proteomic analysis

Gel pieces corresponding to the bands of interest were excised and protein enzymatic cleavage (10 μg) was carried out with trypsin (Promega) 1:20, w/w at 37 °C for 16 h as previously described [41]. Purification and concentration of peptides was performed using C18 Zip Tip Solid Phase Extraction (Millipore). Peptide mixtures were separated by reverse phase chromatography using an UltiMate 3000 UHLPC System (Thermo Fisher Scientific) fitted with an Aurora packed emitter column (Ionopticks). The column temperature was maintained at 40 °C and interfaced online with the Orbitrap Exploris 480 MS. Raw files were processed with MaxQuant [9] v1.6.17.0 using the integrated Andromeda Search engine [10].

### Statistical analyses

All statistical tests and graphs were performed using Prism 6 (GraphPad Software). P < 0.05 was considered significant. One-way ANOVA, Two-way ANOVA with multiple comparisons and two-sided TTEST were employed to compare mean values. Parametric or non-parametric (Mann–Whitney U test) decision was made according Kolmogórov-Smirnov normality test. Tukey correction for multiple comparison was employed (otherwise stated in Figure Legends). The Kaplan–Meier curve was used to estimate the survival function.

## Results

### ATG4B levels correlate with motor neuron survival and control LC3ylation in vivo.

**Fig. 1:**
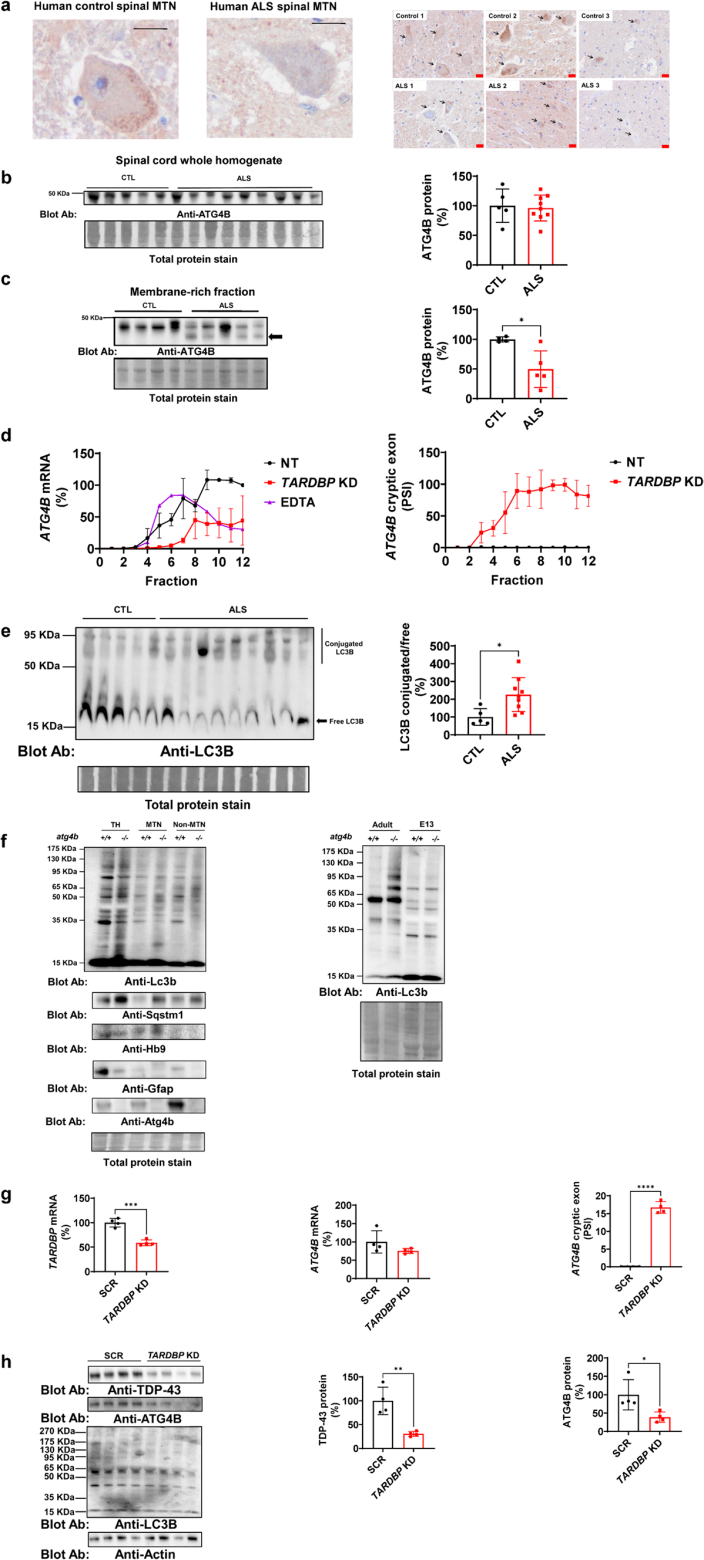
ATG4B function and expression is compromised in ALS. **a** ATG4B immunohistochemistry of human spinal cords demonstrates the enrichment of ATG4B in motor neurons. Left panel shows a higher magnification of a motor neuron in the anterior horn of the lumbar spinal cord of a healthy individual and an ALS patient. Right panel shows a lumbar spinal cord sections from anterior horn from CTL (n = 3) and ALS individuals (n = 3). Arrows point to motor neuron cells. Scale bars indicate 20 µm in length for high- (black labelled scale bar) and low- magnification (red labelled scale bar). **b** ATG4B protein expression analysis in human spinal cord lysates from healthy controls (n = 5) and ALS individuals (n = 9) does not show statistically significant differences. **c** Membrane-rich fractions of human spinal cords where this protein is active exhibit decreased levels in ALS (n = 5) compared with CTL (n = 4). Further, this membrane-rich fraction shows a low molecular weight protein, potentially derived from cryptic exon translation, indicated with an arrow. **d** *ATG4B* mRNA is downregulated in polysomes of *TARDBP* KD HeLa cells and more than 90% of the remaining *ATG4B* in mRNA contains cryptic exons in polysomes (active ribosomes) in *TARDBP* KD cells. Data from three experiments. **e** In line with decreased functionality of ATG4B, one of its substrates, LC3B, shows increased amount in high molecular weight conjugates (60 to 120 kDa) respect to free LC3B in ALS (n = 9) compared with CTL (n = 5) in human spinal cords. **f** Contrary to adult individuals, LC3ylation pattern is not influenced by Atg4b expression during development of mouse spinal cord. However, the loss of Atg4b in motor neurons triggers an accumulation of the autophagy substrate Sqstm1. HB9 is a motor neuron marker. GFAP is an astrocyte marker. Data from three experiment. **g** shRNA *TARDBP* transduction of human iPSC motor neurons efficiently reduces *TARDBP* mRNA, triggering higher levels of cryptic exon splicing in ATG4B transcript (n = 4) compared with scrambled transduction (n = 4). **h** Human iPSC motor neuron *TARDBP* KD (n = 4) express lower levels of ATG4B protein compared with SCR (n = 4). Data shown in graphs are mean values ± SD, *, **, *** and **** indicate, respectively, p < 0.05, p < 0.01, p < 0.001, p < 0.0001 for parametric unpaired two-sided Student’s t-test. Kolmogorov–Smirnov (distance) normality test was assessed. *KD* knockdown. *CTL* control samples. *SCR* scrambled control transduction. *PSI* percentage spliced-in. *MTN* Motor neuron

The consequence of an increased amount in cryptic exon splicing events in *ATG4B* mRNA is its downregulation [47]. Therefore, we proceeded with the quantification of ATG4B protein in ALS, a disease with TDP-43 dysfunction. ATG4B is expressed in human motor neurons (Fig. 1a). ATG4B protein levels in SC lysates are not altered in ALS (Fig. 1b). However, its membrane-bound form is depleted in ALS patients in comparison with healthy individuals (Fig. 1c). Membrane-rich fractions of SC exhibit a lower molecular weight band of ATG4B. We sought to analyse the potential translation of *ATG4B* cryptic isoform that could explain the lower band found in membrane-rich fractions of SC from ALS patients. We isolated polysomes from HeLa *TARDBP* KD cells. *ATG4B* mRNA is mainly localised in polysome fractions associated to active assembled ribosomes (Fraction 6–12) and moved to monosomes upon EDTA addition, with a marked reduction in *TARDBP* KD HeLa cells (Fig. 1d, left panel). More than 90% of polysomic *ATG4B* mRNA contained cryptic exon in *TARDBP* KD HeLa cells **(**Fig. 1d, right panel). In line with decreased functionality of ATG4B, one of its substrates, LC3B, showed an increased LC3ylation ratio, expressing higher levels of high molecular weight conjugates (60 to 120 kDa) normalised by free LC3B (low molecular weight bands) in human ALS SC (Fig. 1e). To study the impact of ATG4B expression in LC3ylation pattern and autophagy specifically in motor neurons, we isolated motor neurons from E13 *atg4b*^*−/−*^ and WT mice embryos. At this stage of the embryogenesis, a higher yield of spinal motor neurons can be extracted [13, 14]. LC3ylation is not altered by the presence of Atg4b at E13 in LSC in any analysed cell fraction (Fig. 1f, left panel, and Fig. s1a, b). However, *atg4b*^*−/−*^ motor neurons contained higher levels of Sqstm1 (Fig. 1f, left panel, and Fig. s1a, ﻿b). HB9 and GFAP were checked to estimate the purification of motor neurons in relation to astroglia. Atg4b protein levels were also checked to demonstrate the lack of expression in KO mice (Fig. 1f, left panel, and Fig. s1a, b) When comparing E13 embryos and adult LSC, LC3ylation is only increased in adults in *atg4b*^*−/−*^ mice, although specific embryonic LC3ylated bands were also observed (Fig. 1f, right panel). We also analysed the effect of *TARDBP* silencing on ATG4B expression in human iPSC motor neurons. We efficiently downregulated *TARDBP* mRNA after lentivirus transduction with shRNA (Fig. 1g, left panel). However, *ATG4B* mRNA did not reach a significant reduction (Fig. 1g, middle panel) although *ATG4B* cryptic exons were included in the 16% of the transcripts (Fig. 1g, right panel). Nevertheless, both TDP-43 and ATG4B protein levels were heavily reduced in these cells after *TARDBP* silencing. LC3ylation, in contrast, was not altered (Fig. 1h).

**Fig. 2:**
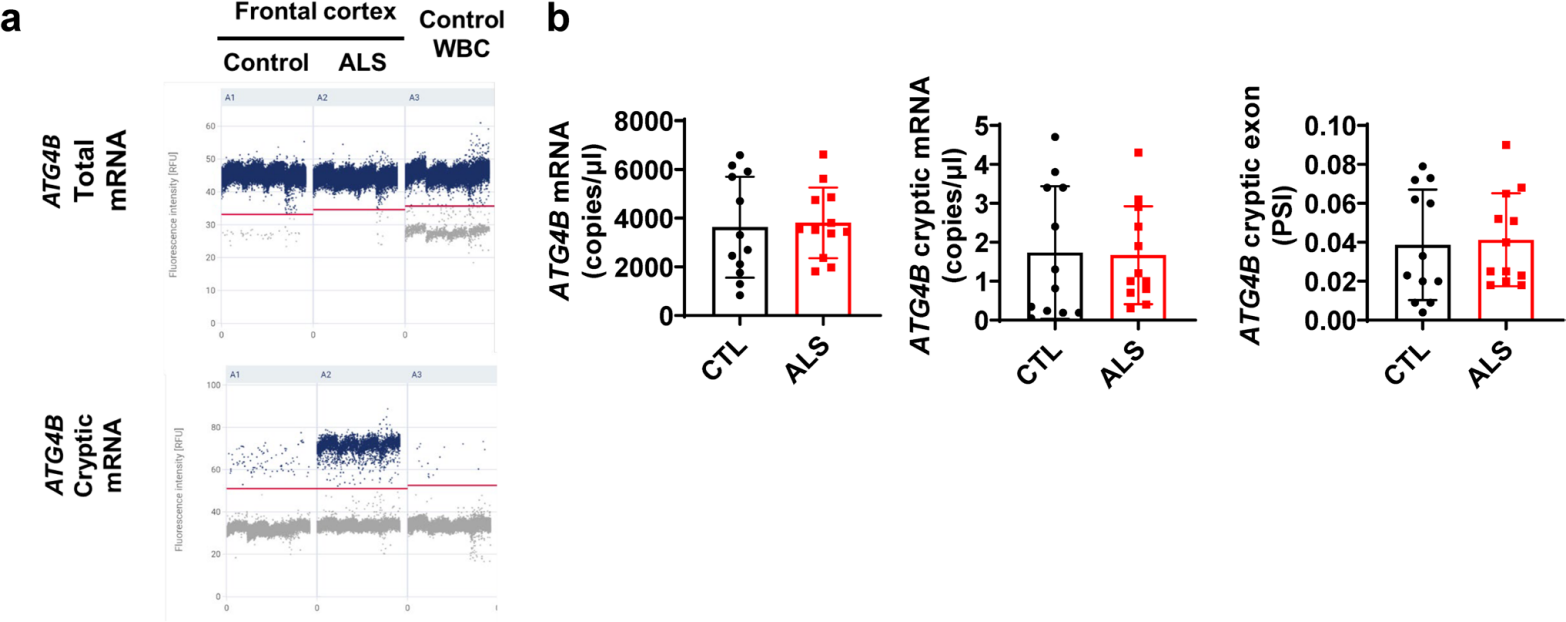
*ATG4B* cryptic exon is expressed in blood cells but does not discriminate ALS cases. **a** dPCR results indicate a good partitioning in frontal cortex and WBC for both total ATG4B expression and cryptic exon of this transcript. *ATG4B* cryptic exon expression is higher in ALS frontal cortex (n = 1) than in control sample (n = 1). **b** In WBC, *ATG4B* expression (left panel), *ATG4B* cryptic exon expression (middle panel) and *ATG4B* cryptic exon PSI (right panel) are not different between CTL (n = 12) and ALS cases (n = 12 Data shown in bar graphs are mean values ± SD. PSI (Percentage Spliced-In). WBC (Whole Blood Cells). Mann Whitney test assessed for statistical analysis

In order to explore the potential use of *ATG4B* cryptic exon as a biomarker of TDP-43 loss of function, we set up a high sensitivity method based on digital PCR (dPCR). We were not able to detect it in peripheral blood cells using RT-qPCR (data not shown). Using dPCR, we detected higher levels of *ATG4B* cryptic exon PSI in an ALS frontal cortex sample (1.97%) than in a control one (0.05%) (Fig. 2a).However, *TARDBP* mRNA expression was not altered (Fig. s2), suggesting that the levels of cryptic exon inclusion do not depend only on TDP-43 expression level but also from other factors (e.g.nuclear localization). Regarding the use in whole blood cells (WBC) RNA, we detected positive partitions for total and cryptic version of *ATG4B,* but neither absolute quantification and cryptic exon inclusion were different between ALS and Controls (Fig. 2b).

### Atg4b deletion dramatically reduces lifespan in a motor neuron disease mouse model

**Fig. 3:**
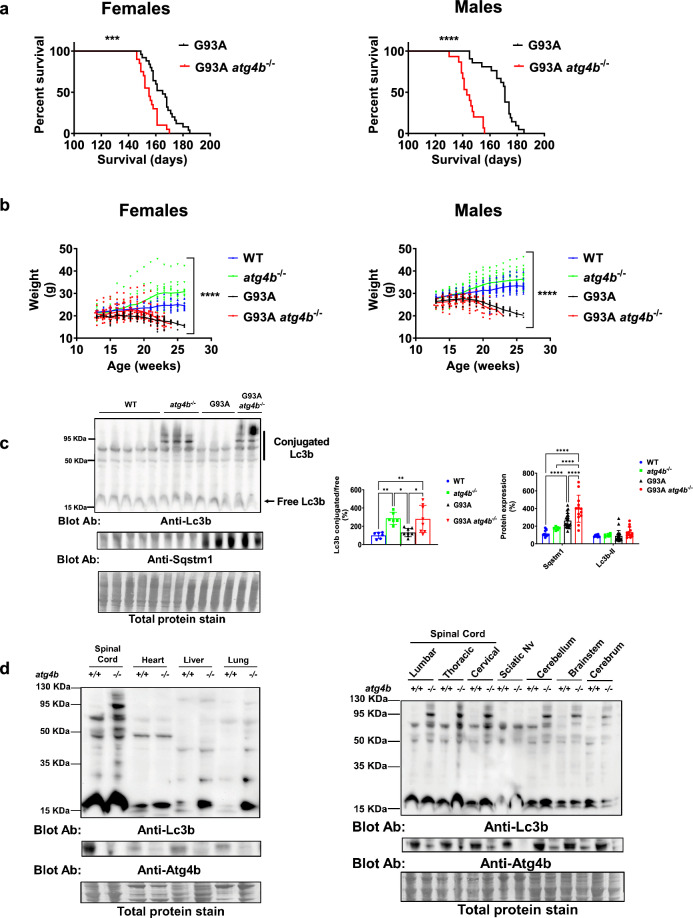
*Atg4b* expression is required for motor neuron survival in the murine model of motor neuron disease hSOD1-G93A mice. **a** Double transgenesis experiments show that G93A *atg4b*^*−/−*^ female mice (n = 20) have a shorter survival than G93A ones (n = 25). This effect is even greater for G93A *atg4b*^*−/−*^ male (n = 15) compared with G93A ones (n = 21). Kaplan-Meyer survival analyses. **b** Disease course as evidenced by weight loss related atrophy of both female (left panel) and male (right panel) is influenced by genotype. Data from females: WT (n = 13), *atg4b*^−/−^ (n = 12), G93A (n = 25), G93A *atg4b*^−/−^ (n = 20). Data from males: WT (n = 27), *atg4b*^−/−^ (n = 20), G93A (n = 18), G93A *atg4b*^−/−^ (n = 15). Two-Way ANOVA test; p-value for genotype (fixed effect type III). **c** *atg4b*^−/−^ genotype is associated to a higher Lc3b conjugated proteins, independently of G93A expression in LSC. Data from WT (n = 6), *atg4b*^−/−^ (n = 6), G93A (n = 8), G93A *atg4b*^−/−^ (n = 8). Bar graphs are mean values ± SD. *, and ** indicate, respectively, p < 0.05, p < 0.01 in One-way ANOVA test and Tukey corrected multiple comparisons. On the other hand, *Atg4b* deletion exacerbates autophagy impairment in LSC from G93A transgenic mice demonstrated by densitometric analyses of Sqstm1 western blot. Data from WT (n = 10), *atg4b*^−/−^ (n = 6), G93A (n = 21), G93A *atg4b*^−/−^ (n = 13). Bar graphs are mean values ± SD. *, **, and **** indicate, respectively, p < 0.05, p < 0.01, p < 0.0001 in Two-way ANOVA test and Tukey corrected multiple comparisons. **d** LC3ylation is also regulated by Atg4b in other central nervous system structures including cerebrum, cerebellum, brainstem and with less intensity in peripheral nervous tissue (sciatic). LC3ylation of peripheral organs such as heart, liver, and lung are not altered by *Atg4b* deletion. Data from three *atg4b*^−/−^ and three WT mice. Sciatic Nv indicates Sciatic nerve

To study the relevance of *Atg4b* and LC3ylation in motor neuron disease, we generated transgenic mice without *Atg4b* expression in the context of an ALS preclinical model G93A. Of note, mouse *Atg4b* mRNA does not contain a cryptic exon controlled by Tdp-43, precluding the use of mutant TDP-43 overexpressing or conditional KO models [28]. For this reason, we used the well-known model of motor neuron disease G93A mouse. Double transgenesis experiments show that *Atg4b* loss impairs the survival (Fig. 3a) and accelerates disease course (Fig. 3b) as evidenced by weight loss of both female and male G93A mice. Notably, there is an increase in LC3ylation ratio that is observed only in *atg4b*^−/−^ mice, independently of G93A transgene (Fig. 3c, left panel). *Atg4b* deletion exacerbated the accumulation of autophagy substrate Sqstm1 in LSC at end-stage of G93A mice without changes in Lc3b-II (Fig. 3c, right panel). Interestingly, deLC3ylation activity of Atg4b is higher in LSC compared with non-neural tissue, including heart, lung, and liver (Fig. 3d, left panel, and Fig. s2a, b). In non-neural tissue, the loss of Atg4b is associated with changes in Lc3b lipidation (low molecular weight) (Fig. 3d, left panel, and Fig. s3a, b). A CNS region-specific LC3ylation was also observed in cerebrum, cerebellum, and brainstem and less marked in sciatic nerve in *atg4b*^−/−^ mice (Fig. 3d, right panel, and Fig. s3c, d).

**Fig. 4:**
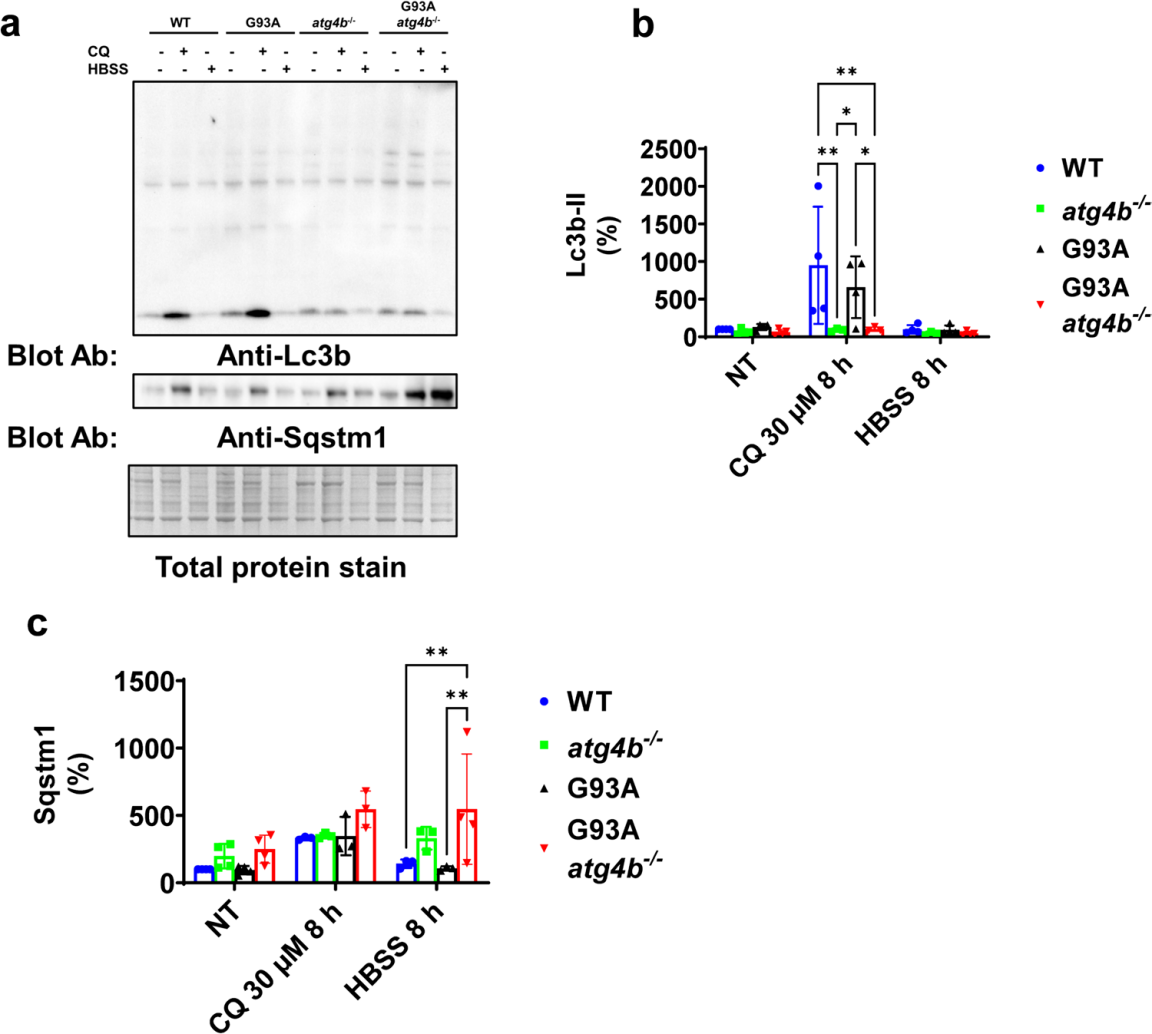
Atg4b is required for Lc3b lipidation in fibroblasts under autophagy inhibition. **a** LC3ylation is not dependent of Atg4b in mouse fibroblast. **b** Lc3b-II accumulation from autophagy inhibition after CQ treatment is not achieved in *atg4b*^−/−^ and G93A *atg4b*^−/−^ genetic background. Data from NT situation: WT (n = 4), G93A (n = 4), *atg4b*^−/−^ (n = 4) and G93A *atg4b*^−/−^ (n = 4); data from CQ treatment: WT (n = 4), G93A (n = 4), *atg4b*^−/−^ (n = 3) and G93A *atg4b*^−/−^ (n = 3); data from HBSS treatment: WT (n = 4), G93A (n = 4), *atg4b*^−/−^ (n = 3) and G93A *atg4b*^−/−^ (n = 4). **c** Sqstm1 is accumulated in G93A *atg4b*^−/−^ under autophagy induction by nutrient starvation with HBSS incubation. Data from NT situation: WT (n = 4), G93A (n = 4), *atg4b*^−/−^ (n = 4) and G93A *atg4b*^−/−^ (n = 4); data from CQ treatment: WT (n = 3), G93A (n = 3), *atg4b*^−/−^ (n = 3) and G93A *atg4b*^−/−^ (n = 3); data from HBSS treatment: WT (n = 3), G93A (n = 3), *atg4b*^−/−^ (n = 3) and G93A *atg4b*^−/−^ (n = 4) Bar graphs are mean values ± SD. * and ** indicate, respectively p < 0.05, and p < 0.01 in. Two-way ANOVA test and Tukey corrected multiple comparisons. *NT* (not treated), *CQ* (Chloroquine), *HBSS* (Hanks’ Balanced Salt Solution)

We aim to explore potential interaction of Atg4b expression and G93A with autophagy stress. For this purpose, we generated an in vitro model based in dermal fibroblasts culture from the different mouse genotypes. Interestingly, LC3ylation was not dependent of Atg4b expression in these cells (Fig. 4a). However, in *atg4b*^−/−^ independently of G93A expression, Lc3b-II did not increase after chloroquine exposure (a lysosomotropic drug which inhibits autophagic degradation in the lysosomes) (Fig. 4b). Furthermore, Sqstm1 was accumulated under starvation in G93A *atg4b*^*−/−*^ fibroblasts (Fig. 4c).

**Fig. 5:**
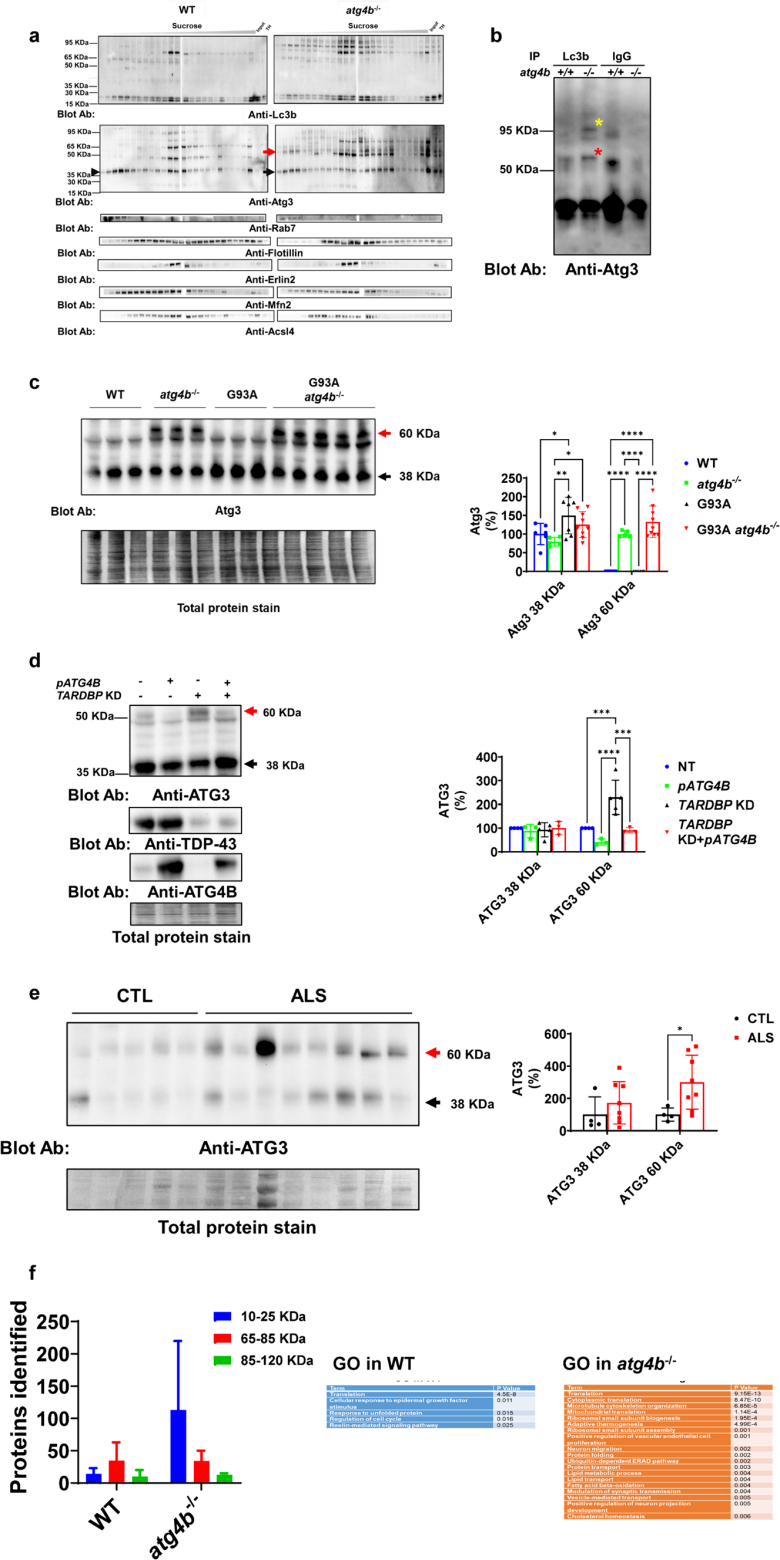
ATG4B regulates ATG3 LC3ylation and its distribution across cell membranes. **a** SC detergent-resistant membranous fractions from *atg4b*^−/−^ mice contain specific high molecular weight bands of Lc3b and Atg3 in different compartments compatible with endosomes and MAMs. Data from a pool of two WT and two *atg4b*^−/−^ mice. **b** Immunoprecipitation experiment using spinal cord lysates from mouse confirms Atg3 as a target of LC3ylation. Data from one WT and one *atg4b*^−/−^ mice. **c** LC3ylated Atg3 (60 KDa) is accumulated in spinal cord from *atg4b*^*−/−*^ mouse and Atg3 (38 KDa) only increases in G93A compares with WT. Data from WT (n = 6), *atg4b*^*−/−*^ (n = 5)*,* G93A (n = 7), G93A *atg4b*^*−/−*^ (n = 9). Two-way ANOVA test and Tukey corrected multiple comparisons. **d** TDP-43 regulates ATG3 LC3ylation by controlling ATG4B levels in HeLa cells. Data from NT (n = 4), *pATG4B* (n = 3), *TARDBP* KD (n = 5), *TARDBP* KD + *pATG4B* (n = 3). Two-way ANOVA test and Tukey corrected multiple comparisons. **e** LC3ylated ATG3 is increased in spinal cord homogenates from human ALS (n = 8) compared with CTL (n = 4). Two-way ANOVA test and Tukey corrected multiple comparisons. **f** Lc3b has many interactors in the spinal cord from *atg4b*^*−/−*^ (n = 2) compared with WT (n = 2). Lc3b co-IP proteins participate in translation, proteostasis and lipid metabolism, among others. Arrows indicate anti-Lc3b and anti-Atg3 blot indicates specific *atg4b*^*−/−*^ 60 KDa band. Bar graphs are mean values ± SD (± SEM in f. *, **, *** and **** indicate, respectively, p < 0.05, p < 0.01, p < 0.001, p < 0.0001 in Two-way ANOVA test and Tukey corrected multiple comparisons. pATG4B indicates plasmid for *ATG4B* overexpression. Red arrows indicate 60 KDa LC3ylated ATG3. Black arrows indicate 38 KDa unmodified ATG3. Yellow asterisk indicates 95 KDa LC3ylated Atg3 in the IP experiment. Red asterisk indicates 60 KDa LC3ylated Atg3 in the IP experiment

To analyse potential changes in LC3ylated proteins in membranous compartments (endosomes, lysosomes, ER, mitochondria and Mitochondria Associated Membranes (MAM)), we performed a fractionation of cell membranes with a sucrose gradient [31]. LC3ylated proteins were enriched in detergent-resistant membranes, mainly found in mitochondria associated membranes (MAM) and endoplasmic reticulum (ER) (Fig. 5a and Fig s4a and s4b). One of the partners of Lc3b closely related to its lipidation and membrane localization is Atg3. An additional 60 KDa band immunoreactive to anti-Atg3 in membranes was found in *atg4b*^*−/−*^ mice LSC. Atg3 from the WT mouse was more enriched in ER/MAM fraction than one from *atg4b*^*−/−*^ KO mouse (Fig. 5a) We analysed endosomal markers (Rab7a) and ER/MAM (Mfn2, Erlin-2, Acsl4 and lotillin) to show the efficiency of the experiment (Fig. 5a and Fig s4a and s4b). To confirm the identity of 60 Kda and 95 Kda LC3ylated proteins, we performed an IP analysis with anti-Lc3b antibody revealing the presence of Atg3 with downstream western blot (Fig. 5b and Fig s4c and s4d). Herein, we wanted to quantify the possible effect of LC3ylation on Atg3 level in LSC from mice. We quantified unmodified 38 Kda and LC3ylated 60 Kda Atg3 expression by western blot. Unmodified Atg3 is increased in G93A but not in G93A *atg4b*^*−/−*^. LC3ylated Atg3 is increased in *atg4b*^*−/−*^ LSC, and its expression was not different compared with G93A *atg4b*^*−/−*^ mice (Fig. 5c). Since *TARDBP* knockdown (KD) resulted in a downregulation of ATG4B protein, a possible dysfunction of this enzyme was analysed. To investigate this hypothesis, we silenced HeLa cells with an shRNA targeting *TARDBP*. The resulting *TARDBP* KD cells expressed a 60 Kda ATG3 band reversed when *ATG4B* is overexpressed (Fig. 5d). We also sought to study this ATG3 modification in human pathology. LC3ylated 60 Kda ATG3, but not unmodified form, is Increased in ALS SC **(**Fig. 5e). Other LC3ylated proteins are predicted based on multiple bands in anti-LC3B blots. To identify them, we performed a proteomic analysis of LC3B immunoprecipitated proteins after a separation in polyacrylamide gel (25 kDa = range 0–25 kDa; 60 kDa = range 60–85 kDa; 85 kDa = range 85–120 kDa) to increase technique resolution. A higher number of proteins associated with Lc3b were found in *atg4b*^*−/−*^ LSC (Fig. 5f, left panel), associated with multiple functions (Fig. 5f, right panel). Protein IDs from co-IP experiments are listed in Table s1.

### Antisense oligonucleotides targeting *ATG4B* cryptic exon restore its mRNA levels

**Fig. 6:**
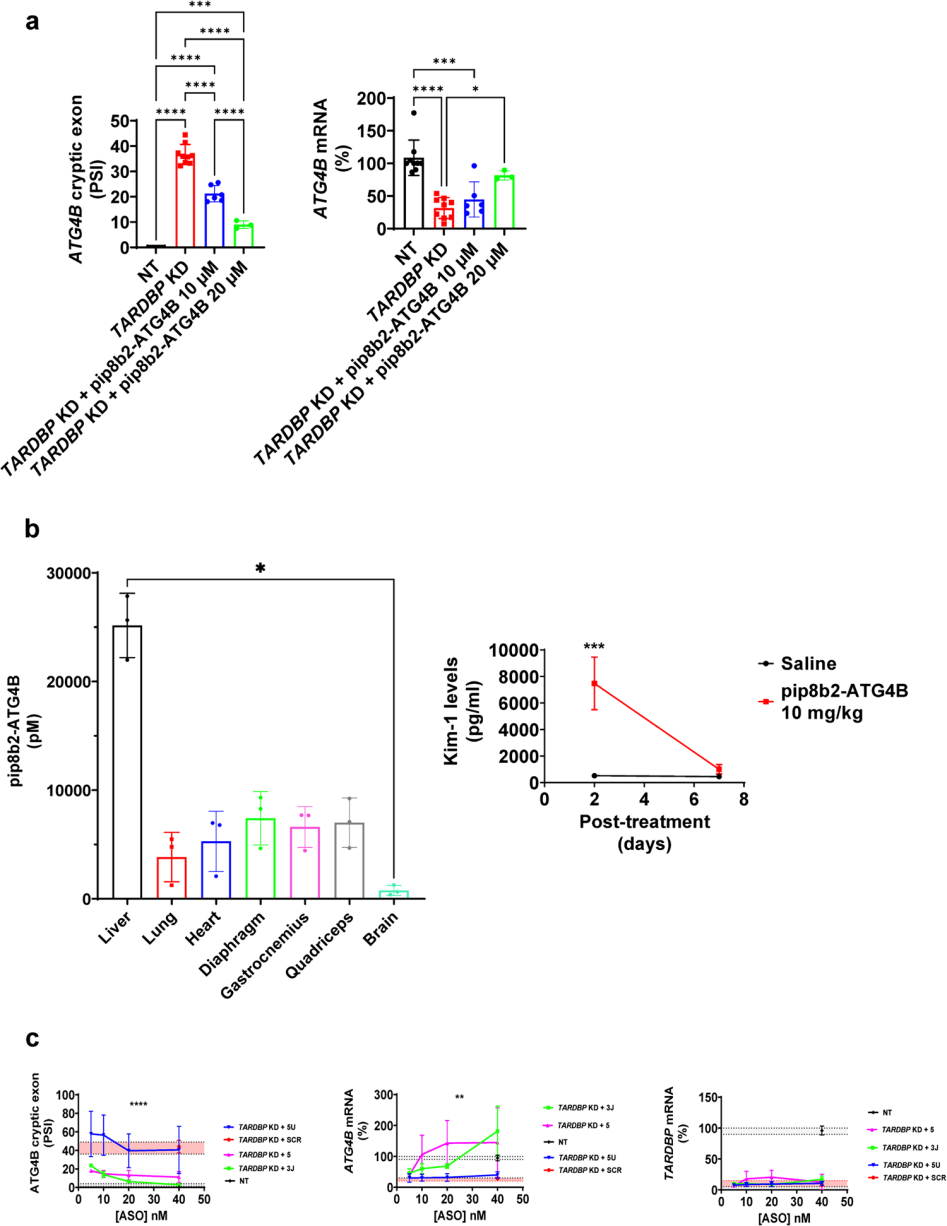
ASOs targeting cryptic exon splicing rescue *ATG4B* expression. **a** In *TARDBP* KD HeLa cells, the use of a specific antisense oligonucleotide against ATG4B cryptic exons (pip8b2-ATG4B) employing PMO chemistry prevents *ATG4B* cryptic exon splicing and restores mRNA expression. Data from NT (n = 9), *TARDBP* KD (n = 9), *TARDBP* KD + pip8b2-ATG4B 10 µM (n = 6), *TARDBP* KD + pip8b2-ATG4B 20 µM (n = 3). Graphs are mean values ± SD. *, *** and **** indicate, respectively, p < 0.05, p < 0.001, p < 0.0001 in Two-way ANOVA test and Tukey corrected multiple comparisons. Two-way ANOVA test and Bonferroni corrected multiple comparisons. **b** Furthermore, this antisense oligonucleotide reaches brain tissue after IV injection (n = 3). Interestingly, though at 2 days signs of renal toxicity –evidenced by high Kim-1 levels- were present, this was normalized 7 days after the injection (n = 5). * represents p < 0.05 in One-way ANOVA Kruskal–Wallis test and Dunn’s corrected multiple comparisons for biodistribution analysis. In the case of Kim-1 levels, *** indicates p < 0.001 individual comparison in 24 h after Two-way ANOVA test and Bonferroni's multiple comparisons test. Graphs are mean values ± SD. *, and *** indicates, respectively, p < 0.05, p < 0.001. (**c**) The rescue effect of LNA mixmer ASOs is evident at lower concentrations (nM scale) targeting 3’ splice junction (3 J) and TDP-43 biding sequence (5) of *ATG4B* cryptic exon reducing cryptic exon splicing. ASOs do not alter *TARDBP* mRNA expression. Data from NT (n = 3), *TARDBP* KD + SCR (n = 3), *TARDBP* KD + 3 J (n = 3), *TARDBP* KD + 5 (n = 3) and *TARDBP* KD + 5U (n = 3). Graphs are mean values ± SD. ** and **** indicate, respectively, p < 0.01, p < 0.0001 in Two-way ANOVA test for ASO treatment effect. Red range indicates expression levels in *TARDBP* KD + SCR. Gray range indicates expression levels in NT

We aimed to explore how splice switching ASOs could prevent *ATG4B* cryptic splicing, thus restoring its normal expression. We designed specific ASOs targeting *ATG4B* splice sequence regulators such as 3’ splice junction and TDP-43 binding region (Fig. s5). In *TARDBP* KD HeLa cells, the use of a specific P-PMO ASO against TDP-43 binding sequence from *ATG4B* cryptic exon (pip8b2-ATG4B) prevented in a dose-dependent its splicing (Fig. 6a, left panel), leading to a recovery in the canonical form of *ATG4B* mRNA (Fig. 6a, right panel). Further, this ASO reached brain tissue at nM scale 2 weeks after the IV administration (tail vein injection) of 10 mg/kg (Fig. 6b, left panel). The treatment induced reversible kidney toxicity as measured by Kidney Injury Molecule 1 (kim-1) levels in urine which was normalized seven days after the injection (Fig. 6b, right panel). Likewise, negatively charged LNA mixmer ASOs targeting 3’ splice junction (ASO 3 J) and TDP-43 binding sequence (ASO 5) also reduced cryptic exon splicing (Fig. 6c, left panel) and restored *ATG4B* mRNA levels in *TARDBP* KD HeLa cells (Fig. 6c, middle panel) without altering *TARDBP* expression (Fig. 6c, right panel).

**Fig. 7:**
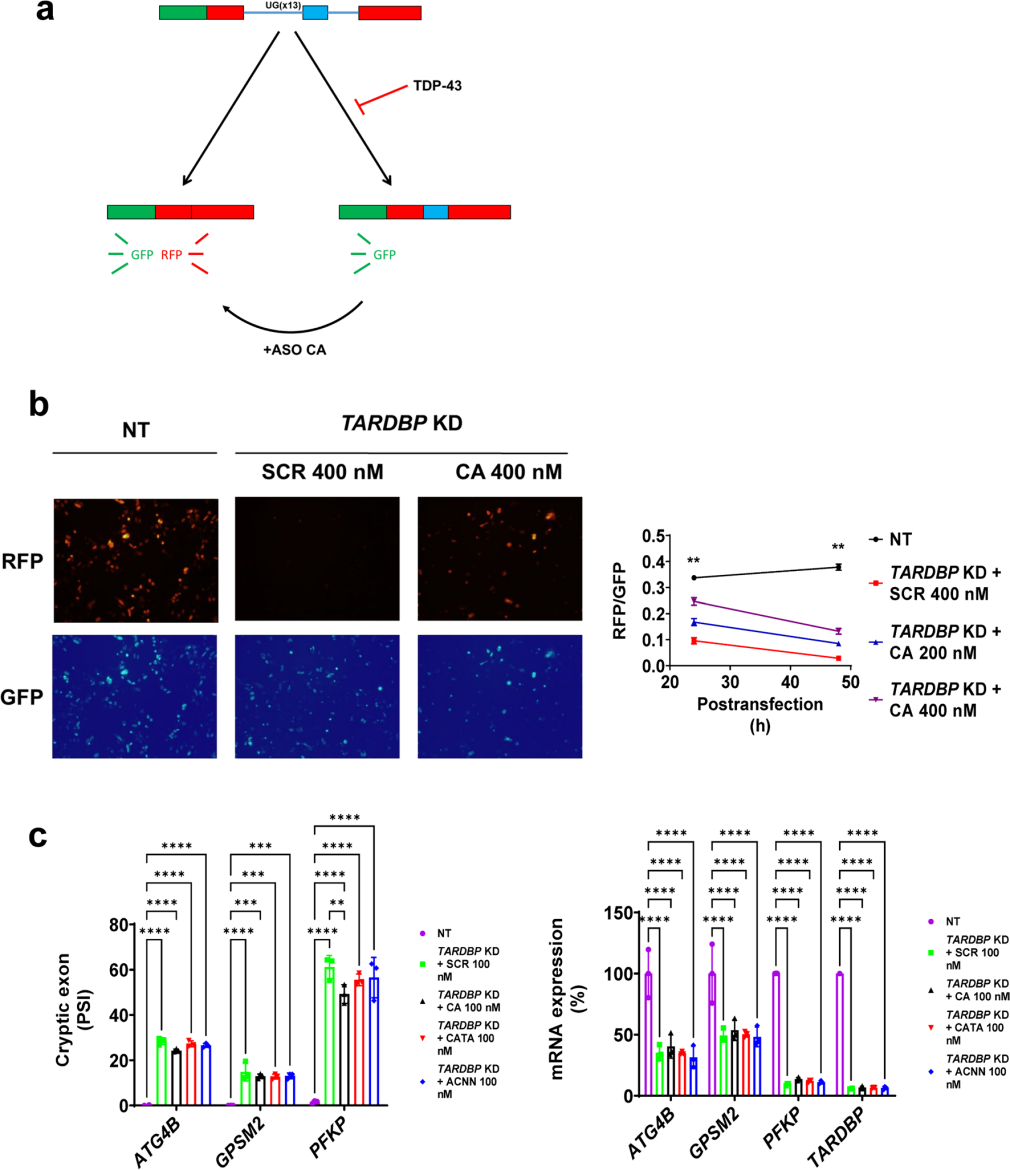
ASO composed by repetitive CA sequence can restore the correct splicing of TDP-43 mini-gene reporter in *TARDBP* KD cells. **a** A dual reporter mini-gene assay of TDP-43 splicing function. **b** ASO CA can partially rescue the control situation in *TARDBP* KD HeLa cells. Data from 24 h post-transfection: NT (n = 204 cells), *TARDBP* KD + SCR 400 nM (n = 114 cells), *TARDBP* KD + ASO CA 200 nM (n = 116 cells), *TARDBP* KD + ASO CA 400 nM (n = 156 cells). Data from 48 h post-transfection: NT (n = 254 cells), *TARDBP* KD + SCR 400 nM (n = 147 cells), *TARDBP* KD + ASO CA 200 nM (n = 235 cells), *TARDBP* KD + ASO CA 400 nM (n = 196 cells). Graphs are mean values ± SD. ** indicates a minimum p < 0.01 in Two-way ANOVA test and Tukey corrected multiple comparisons for all combination in each post-transfection time. (**c**) Multitarget ASOs does not rescue cryptic exon splicing of selected genes. Data from NT (n = 3), *TARDBP* KD + SCR 100 nM (n = 3), *TARDBP* KD + ASO CA 100 nM (n = 3), *TARDBP* KD + ASO CATA 100 nM (n = 3), *TARDBP* KD + ASO ACNN (n = 3). Bar graphs are mean values ± SD. **, *** and **** indicate, respectively, p < 0.01, p < 0.001, p < 0.0001 in Two-way ANOVA test and Tukey corrected multiple comparisons

Finally, we explored the possibility of generating multitarget ASOs complementary to more than one TDP-43 binding site. To do that, we designed ASOs targeting the TDP-43 consensus motif (UG repeats) [3]. Oligo CA is composed by 8 repeats of CA, complementary to continuous UG repeats. Since human cryptic exons are not always continuous UG repeats [17], we also designed oligo CATA and a mix of ASOs (oligo ACNN) targeting not continuous UG repeats (see Table 1 for details). We took advantage of *CFTR* exon 9 fluorescent mini-gene assay to measure TDP-43 function [39]. In this case, *CFTR* exon 9 splicing has a continuous UG repeat with a perfect complementary binding to oligo CA. Red fluorescent depends on TDP-43 function, while green fluorescence is always spliced in mRNA (Fig. 7a). Red fluorescence was reduced upon *TARDBP* silencing in HeLa cells and rescued with oligo CA (Fig. 7b). However, multitarget ASOs were not effective preventing cryptic exon splicing of genes with non-continuous UG repeat or not fully complementary to CATA or ACNN, including *ATG4B*, *GPSM2* and *PFKP* (Fig. 7c).

## Discussion

In this study, we demonstrate ATG4B dysfunction in ALS and propose novel therapeutic agents based on this.

Regarding ATG4B expression and ALS, we previously demonstrated a role in autophagy flux in *TARDBP* KD models [47]. Our current work furthers this understanding, revealing nuanced insights into the interplay between ATG4B and autophagy in ALS pathology, suggesting potential avenues for therapeutic interventions targeting ATG4B-mediated pathways. To further characterize this interaction in other primary cell lines and in vivo we generated a G93A *atg4b*^−/−^ model and analyzed autophagy markers in different tissues and in primary fibroblasts. Our data suggests that ATG4B is essential for the clearance in SQSTM1 containing cargoes (mainly protein aggregates), through LC3B processing, at end-stage of the disease. This is crucial as it underlines the role of ATG4B in maintaining cellular homeostasis and its potential implication in the progression of neurodegenerative diseases. That indicates a loss of compensatory mechanisms during disease evolution, which is exacerbated in the absence of ATG4B. This loss is noteworthy the critical balance maintained by ATG4B and its impact on cellular health in the context of ALS. Our results using primary fibroblasts from mice highlights the critical role of Atg4B in the formation of Lc3b-containing vesicles [22, 56]. Atg4b loss impairs the formation of Lc3b-containing vesicles during a late autophagy flux inhibition with a lysomotropic agent (chloroquine) and reduces the effect of starvation on autophagy activation. It is therefore reasonable to think that the loss of ATG4B triggers an autophagy impairment and ultimately the accumulation of damaged organelles and protein aggregates which could further promote cellular toxicity. In probing the functions of ATG4B, our examination of its role in deLC3ylation of protein revealed a comprehensive landscape of its interactions and functions. Stable covalent complexes with LC3B (LC3ylation) represent a recently described post-translational modification (PTM) in vitro [1]. However, we are the first to describe this PTM in vivo and its association with ALS and TDP-43 proteinopathies. These findings are significant as they expand the current understanding of ATG4B’s diverse roles within the cell and its potential implications in proteinopathies such as ALS. LC3ylation pattern is variable among tissues and ATG4B might have different impact on them. Our data demonstrates a predominant role for deLC3ylation function of ATG4B in CNS (but not in peripheral nervous system). This function is mainly relevant in adult individuals, suggesting compensatory mechanisms during embryogenesis, although some specific bands are only present in embryos. Our data indicate that ATG4B function is critical to sustain autophagy in motor neurons. Interestingly, *Atg4b* deletion results in a defective Lc3b priming and lipidation in adult peripheral tissues, with minimal effect in LC3ylation. This uncovers a cell-specific predominant role of ATG4B. Consequences of a lack of deLC3ylation activity in CNS are unknown but they are likely associated with ALS in mouse and human. One of the known targets of LC3ylation is ATG3 [1]. Our data confirms LC3ylation of ATG3 in mouse and human CNS tissue and in cell lines after TDP-43 silencing, which is rescued by ATG4B re-expression. This confirms that LC3ylation is a conserved PTM, regulated in humans by TDP-43 repression of ATG4B cryptic exon splicing.

We thought that LC3B conjugation in CNS might consume unmodified ATG3. G93A transgene expression in LSC triggers a higher expression of Atg3, potentially linked to an increase on autophagy demand. Nevertheless, this mechanism is lost in the absence of Atg4b and, in turn, a specific LC3ylated Atg3 is accumulated, potentially consuming and inhibiting unmodified Atg3 induction. Since ATG3 participates in the early steps of autophagy during autophagosome formation, we hypothesize that ATG3 modification could change its distribution across intracellular membranes. This is the case of ATG3 acetylation that directs this protein to the endoplasmic reticulum [27]. The presence of ATG4B induces an enrichment of ATG3 in ER/MAM fraction, in contrast to its absence, which leads to ATG3 enrichment mainly in the endosomal fraction. A reduced expression of this protein in this membrane could disturb the formation of autophagosomes [54]. We also explore the potential wide effect of ATG4B on LC3B interactions with other proteins. Our data indicates that ATG4B activity counteracts the interaction of LC3B with hundreds of proteins involved in a diversity of processes, not only autophagy, such as protein translation, ubiquitin-dependent ER associated degradationpathway and lipid metabolism.

Regarding the biomarker potential of *ATG4B* cryptic exon, and although the inclusion of the cryptic exon in *ATG4B* mRNA is predicted to trigger NMD [28], we detected a truncated band in human ALS samples compatible with cryptic translation. Notably, cryptic proteins derived from the translation of mRNA containing cryptic exons are recently described as potential valuable biomarkers [40]. Cryptic exon expression is highly dependent of cell type [21]. Therefore, experimental validation on human motor neurons is necessary to support potential therapies targeting these cells. Here we demonstrated the presence of ATG4B cryptic exon in human iPSC motor neurons after TDP-43 silencing. It highlights the sensibility of this gene in response to downregulation of TDP-43 expression. Interestingly, total *ATG4B* mRNA levels are not altered (25% of reduction, not statistically significant) but 16% of the total mRNA are not full-transcript and potentially do not produce functional protein. High cryptic exon inclusion with a lower impact in mRNA levels reflects a low capacity of degrading NMD substrates, that can lead to accumulation of truncated protein. Notably, a great reduction is observed in ATG4B protein levels in human iPSC motor neurons, compromising its function. However, LC3ylation remains unchanged. These cells are differentiated to motor neurons for 6 days and potentially they do not display age-dependent changes [42]. Like mouse primary embryonic motor neurons from *atg4b*^−/−^ that do not accumulate LC3ylated proteins in LSC, aging could be important for the accumulation of LC3ylated proteins. Aging is an important risk factor in ALS [34] and cellular senescence is present in human patients and animal models [45, 46, 49, 51].

Moreover, in this work we evidence the presence of cryptic *ATG4B* in polysomes, indicative of active translation and the potential location of this protein product in the membranous compartment in ALS patients. Another potential biomarker is the detection of mRNA containing the cryptic exon in fluids. The use of dPCR enables a greater dynamic range and a single-molecule threshold, making it an excellent solution for low abundant RNAs, as in the case of cryptic exons. Our data in frontal cortex is very similar to what was previously reported using qPCR [47], confirming by an alternative method an inclusion rate close to 2% in ALS and almost 100X lower in controls. In this work we confirm a very low abundance of *ATG4B* cryptic exon in comparison to total *ATG4B* mRNA in blood cells, suggesting that TDP-43 is functioning properly in this tissue. However, cryptic exons have a different splicing dependency of TDP-43 dose-dependent splicing. *STMN2* cryptic exon, for instance, is spliced-in after a minimal loss of TDP-43 function [40], suggesting that it is a sensitive biomarker in non-neural tissue. Further studies will test the potential use of dPCR for *ATG4B* and *STMN2* to analyse CSF and plasma exosomes, containing potential vesicles derived from diseased neurons.

In this work, we propose a novel solution to rescue ATG4B function triggered by TDP-43 loss. Our ASOs target a specific human splicing event that is present in ALS [47], FTLD-TDP-43 [28] and Alzheimer’s Disease [44] but not in people without TDP-43 proteinopathies. The correction of *ATG4B* cryptic splicing in human cells shown in a dose-dependent manner in this study has the potential to become a treatment across multiple patient populations with neurodegenerative diseases. Similar pharmacokinetic properties of therapeutic compounds using this chemistry were associated with phenotype corrections in myotonic dystrophy [23] and spinal muscular atrophy [15]. We have demonstrated a cryptic exon splicing inhibition using ASOs with different chemistry: LNA mixmer and P-PMO. Pip8b2-ATG4B is mainly accumulated in liver but a little amount can cross the blood–brain barrier to reach CNS after intravenous administration. It could be relevant in clinical trials because intrathecal administration is a highly invasive approach. Moreover, this P-PMO is not likely to cause irreversible kidney toxicity measured by kim-1 levels in urine. Kim-1 is a transmembrane glycoprotein in renal proximal tubules [18]. After a renal injury, its extracellular domain is released from membrane triggering an increase in urinary levels [50]. Kim-1 is a Food and Drug Administration (FDA) and European Medicines Agency (EMEA) approved injury biomarker of kidney toxicity [50].

ASOs targeting 3′ splice junction can rescue *ATG4B* expression in *TARDBP* KD cells. Moreover, ASOs targeting TDP-43 binding sequence of *ATG4B* (like pip8b2-ATG4B and ASO 5) also inhibit cryptic splicing, thus mimicking the effect of TDP-43. This suggests that the mere presence of TDP-43 or ASO is enough to sterically prevent the recognition of a cryptic splice site. Therefore, ASOs targeting the consensus sequence of TDP-43 RNA binding (multi-target) could inhibit a larger number of cryptic exons. Notably, our results indicate that ASO CA inhibits splicing of genes with UG tracts like in *CFTR* exon 9 [2, 16]. The development of multitarget ASOs remains crucial, as with refinement, they have the potential to correct the missplicing of multiple TDP-43 repressed exons. A transcriptomic assay would be useful to uncover the potential use of multitarget ASOs for TDP-43 proteinopathies.

The main limitation of our study is the lack of pre-clinical efficacy evaluation of our ASOs. To overcome this issue, we envisage the use of humanized mouse models carrying *ATG4B* cryptic exon in *Tdp-43* conditional KO context. Such experiments will provide insights into the translation of our findings from cellular models to whole organisms, evaluating the real-world potential of our therapeutic strategy. In conclusion, ATG4B functions are compromised in ALS, worsening disease progression, and can be recovered with our ASOs.

## Supplementary Information

Below is the link to the electronic supplementary material.

Supplementary file1 Figure s1. Additional experiments of LC3ylation and sqstm1 in E13 mouse spinals cords, and purified motor neurons (TIFF 1327 kb)

Supplementary file2 Figure s2. TARDBP mRNA quantification of ALS samples from human frontal cortex. Not-significant differences after a parametric unpaired two-sided Student’s t-test. Kolmogorov-Smirnov (distance) normality test was assessed (TIFF 2948 kb)

Supplementary file3 Figure s3. Additional experiments of LC3ylation in mouse CNS regions and peripheral tissues (TIFF 2430 kb)

Supplementary file4 Figure s4. Additional experiments of membrane fractionation and immunoprecipitation (TIFF 10797 kb)

Supplementary file5 Figure s5. Schematic position of ASOs targeting ATG4B pre-mRNA. Image modified from NCBI (TIFF 3327 kb)

Supplementary file6 (XLSX 27 kb)

## Data Availability

Data will be provided by the authors on reasonable request basis.

## Abbreviations

ALS: Amyotrophic lateral sclerosis
ASO: Antisense oligonucleotide
CQ: Chloroquine
E13: Embryonic day 13
ER: Endoplasmic reticulum
GFP: Green fluorescent protein
HBSS: Hanks’ balanced salt solution
KD: Knockdown
LSC: Lumbar spinal cord
MAP1LC3/LC3: Microtubule associated protein 1 light chain 3
MAM: Mitochondrial-associated membranes
nM: Nanomolar
NMD: Non-sense mRNA -mediated decay
NT: Not treated
PBS: Phosphate-buffered saline
P-PMO: Peptide phosphorodiamidate morpholino oligomer
RFP: Red fluorescent protein
SC: Spinal cord
SCR: Scrambled
SD: Standard deviation
shRNA: Short hairpin RNA
WBC: Whole blood cells
WT: Wild-type
μl: Microliter
μg: Microgram
μm: Micrometre
μM: Micromolar
